# Supplementary material for: Specialized transendothelial dendritic cells mediate thymic T-cell selection against blood-borne macromolecules
Source: Nat Commun. 2021 Oct 28;12:6230. doi: 10.1038/s41467-021-26446-x (PMC8553756; doi:10.1038/s41467-021-26446-x)
Supplement: Supplementary file 1 — Supplementary Information [file 41467_2021_26446_MOESM1_ESM.pdf]

Supplementary Information

Specialized transendothelial dendritic cells mediate thymic T cell selection against blood-borne macromolecules

Elisabeth H. Vollmann[1,6], Kristin Rattay[1,2], Olga Barreiro[1], Aude Thiriot[1], Rebecca A. Fuhlbrigge[1], Vladimir Vrbanac[3,7], Ki-Wook Kim [4, 8], Steffen Jung[4], Andrew M. Tager[3,] and Ulrich H. von Andrian[1,5]

[1] Department of Immunology & HMS Center for Immune Imaging, Harvard Medical School, Boston, MA 02115, USA

[2] Institute of Pharmacology, Biochemical Pharmacological Center, University of Marburg, 35032 Marburg, Germany

[3] Massachusetts General Hospital, Boston, MA 02114, USA

[4] Department of Immunology, Weizmann Institute of Science, Rehovot, Israel

[5] Ragon Institute of MGH, MIT and Harvard, Cambridge, MA

[6] Current address: Merck Research Laboratories, Boston, MA 02115

[7] Current address: Massachusetts General Hospital, Humanized Immune System Mouse Program (HISMP), Boston, MA 02114, USA

[8] Current address: Department of Pharmacology and Regenerative Medicine, University of Illinois College of Medicine, Chicago, IL 60612, USA

Corresponding author:

Ulrich H. von Andrian, MD., Ph.D.

Department of Immunology, Harvard Medical School, Boston, MA 02115, USA.

E-mail: uva@hms.harvard.edu

phone: +1-617-432-6827

fax: +1-617-432-6829

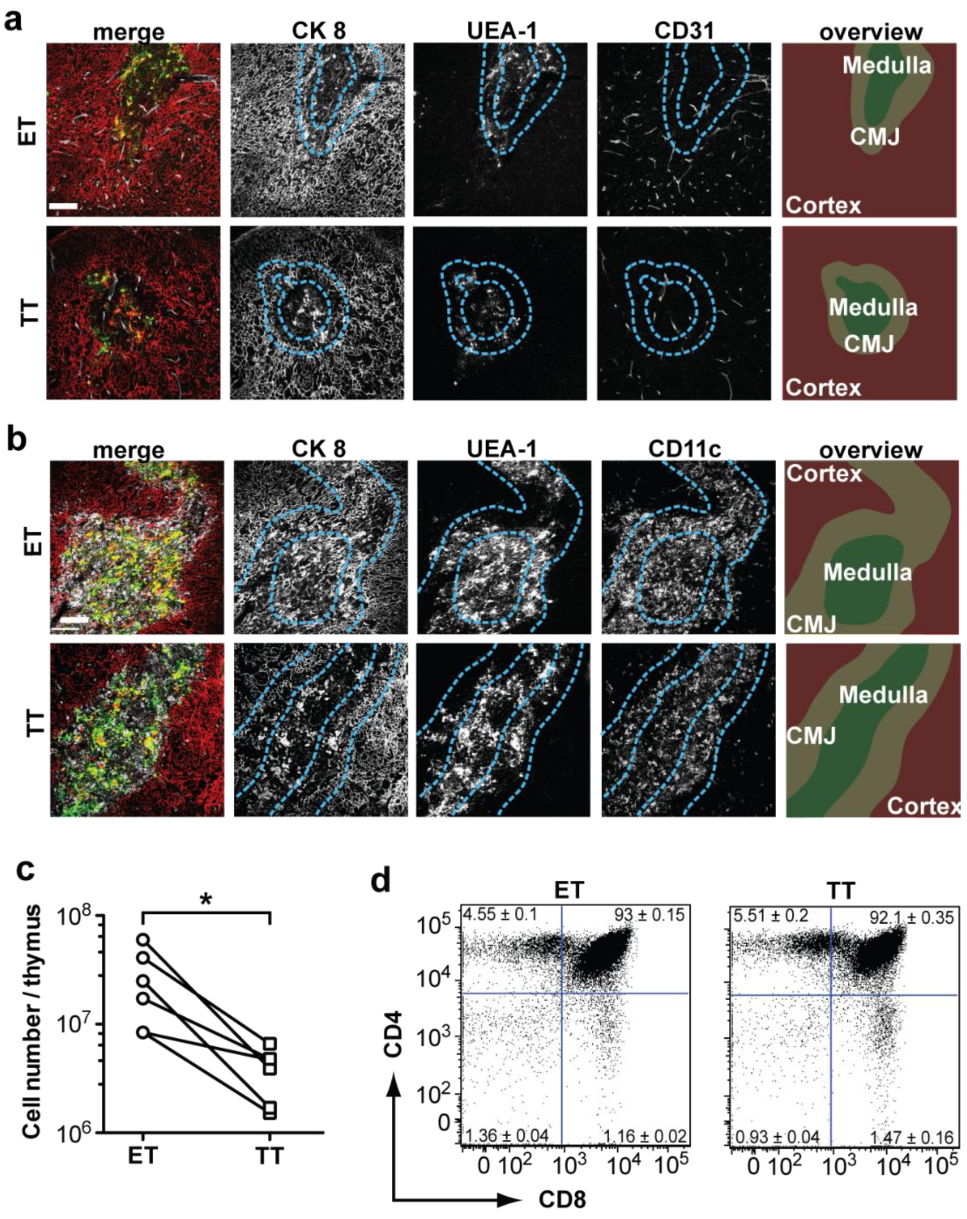

Supplementary Fig. 1 Comparison of endogenous and transplanted thymi.

Fetal thymi were transplanted under kidney capsules of recipient mice and endogenous thymi (ET) and transplanted thymi (TT) were analyzed 4-9 weeks later. (a and b) Cryosections of ETs and TTs were stained with anti-cytokeratin 8 mAb (CK8, red, a and b), the lectin UEA-1 that specifically binds to mTECs (green, a and b) and mAbs against CD31 (white, a) or CD11c (white, b). Cortex, medulla and cortex-medulla-junction (CMJ) were identified according to the cytokeratin 8 and UEA-1 staining (Overview, a and b). These images were used to assess the density of $CD11c^+$ and $CD31^+$ area in the cortex, CMJ and medulla (Fig. 1a and d). n = 5 experiments for each staining, shown are representative confocal micrographs. (c) Absolute cell numbers of the endogenous and transplanted thymi 4 weeks after thymus transplantation. Each pair of symbols represents an individual animal. n = 6 (two-tailed, paired Student's t-test, *: P = 0.0374). (d) Representative FACS plots show the CD4-CD8 profile of thymocytes from ETs and TTs 4 weeks after transplantation.

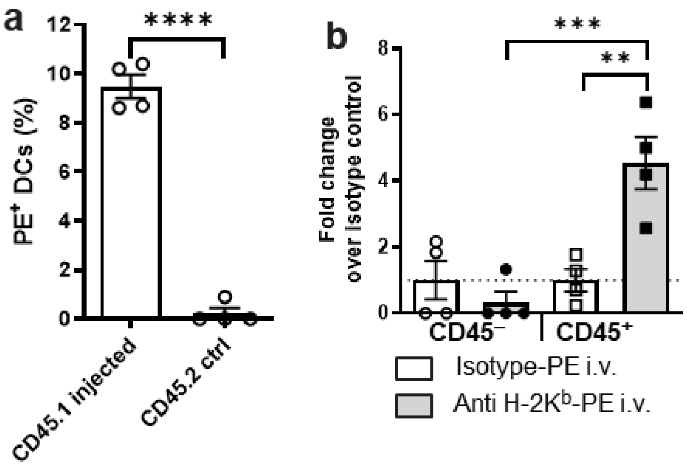

Supplementary Fig. 2 Validation of the in vivo PE$^+$ labeling.

(a) CD45.1 mice were injected with anti-CD11c-PE and euthanized 2 minutes later while CD45.2 mice were left untreated. Single cell suspensions of the thymi of two CD45 congenic mice each were prepared in the same dish and PE$^+$ DCs among CD45.1$^+$ and CD45.2$^+$ cells were analyzed by flow cytometry. (b) C57Bl/6 mice were injected i.v. with anti-H-2Kb-PE or isotype-PE and sacrificed 2 minutes later. CD45$^+$ and CD45$^-$ cells were evaluated for acquisition of the PE label. Data is expressed as fold change of PE$^+$ cells over isotype-PE control. Data are representative of 2 (a) and 3 (b) independent experiments with 5 mice per group. P values were calculated using unpaired, two- tailed Students t-test (a) and one-way ANOVA (b). ****P < 0.0001, ***P = 0.007, **P = 0.003, n.s. not significant; mean ± s.e.m.

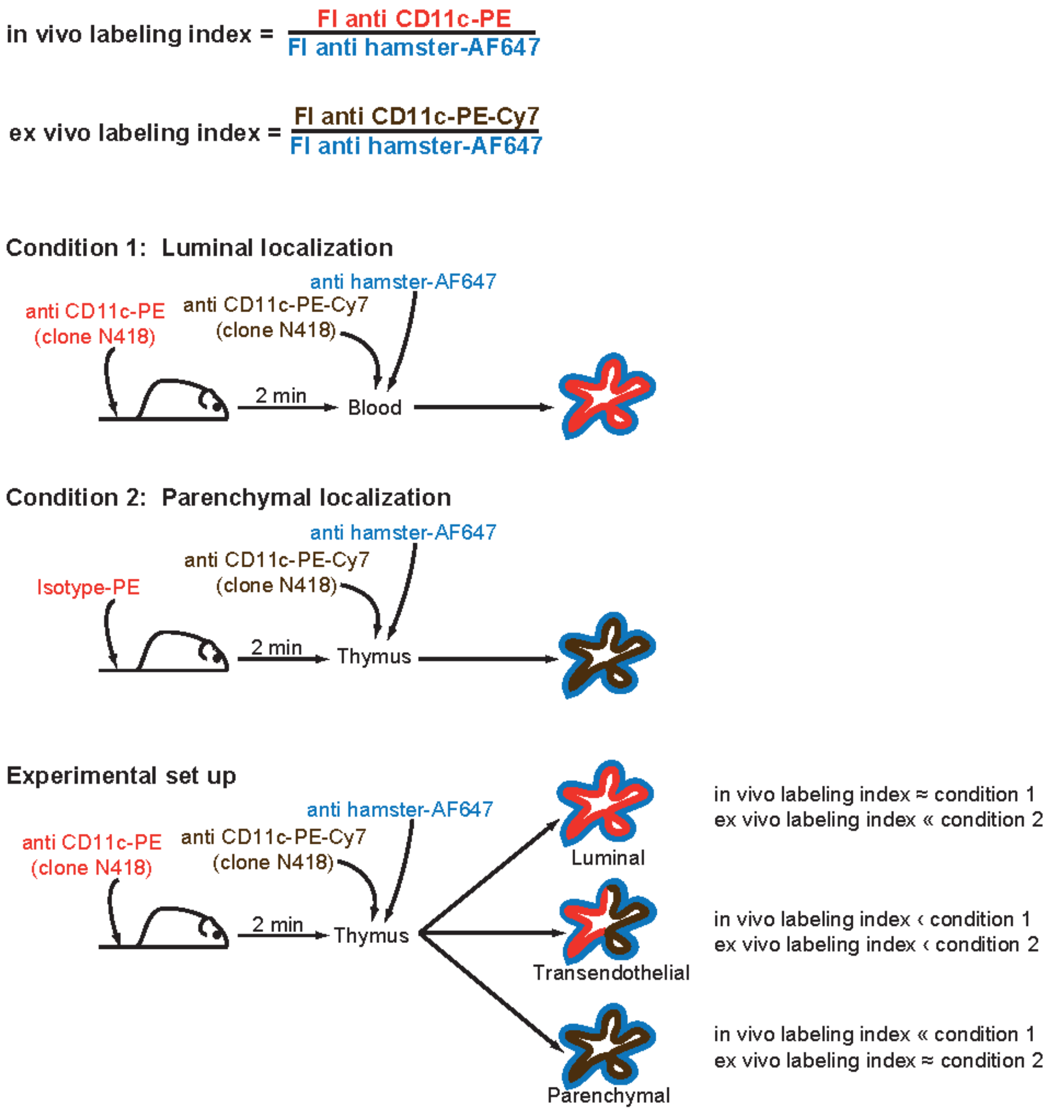

Supplementary Fig. 3 Schematic of competitive CD11c staining to identify cell localization.

Staining indexes were determined by applying a competitive staining protocol whereby mice were injected with anti-CD11c-PE (clone N428) IV, sacrificed 2 minutes later and single cell suspensions were stained ex vivo with anti-CD11c-PE-Cy7 (clone N418) and anti-hamster-AF647. In vivo and ex vivo staining indexes were calculated by dividing each cell's fluorescence intensity (FI) for the respective in vivo (PE) or ex vivo stain (PE-Cy7) by the FI of anti-hamster-AF647 to normalize for differences in the overall expression of CD11c. Lu-DCs are identified PE^{+} $PE\text{-}Cy7^{-}$, Par-DCs are PE– $PE\text{-}Cy7^{+}$and TE-DCs have an intermediate phenotype. Blood borne DCs can be used to identify Lu-DCs (Condition 1) and isotype-PE injected thymic DCs can act as a surrogate for Par DCs (Condition 2). With this gating strategy one can then identify Lu-DCs, Par-DCs and TE-DCs (Experimental setup).

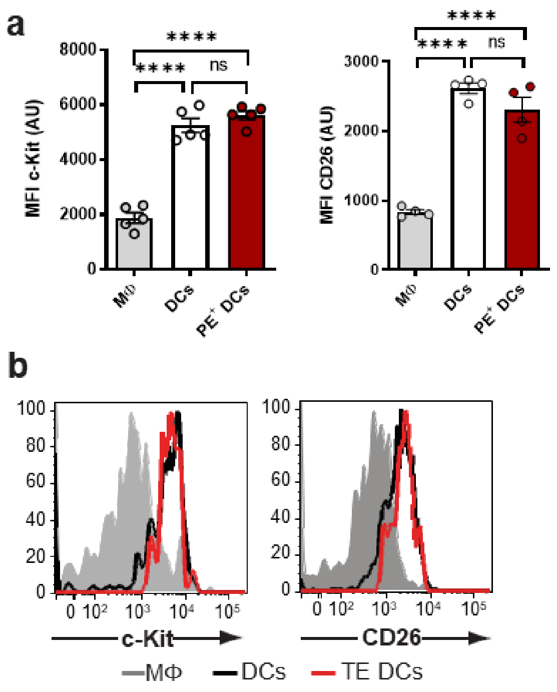

Supplementary Fig. 4 Analysis of thymic macrophages and DCs

(a and b) Thymic macrophages (Mϕ), parenchymal denritic cells (Par-DCs) and PE+ DCs were evaluated for c-Kit (left panel) and CD26 (right panel) expression. (a) mean fluorescence intensities (MFI) of each cell population, (b) Representative FACS histogram; TE DCs: transendothelial dendritic cells, n=3 independent experiments with 5 mice/each for c-Kit and 4 mice/each for CD26 (one-way ANOVA). Bars and error bars represent mean ± SEM. ****: $P < 0.0001$, n.s.: not significant

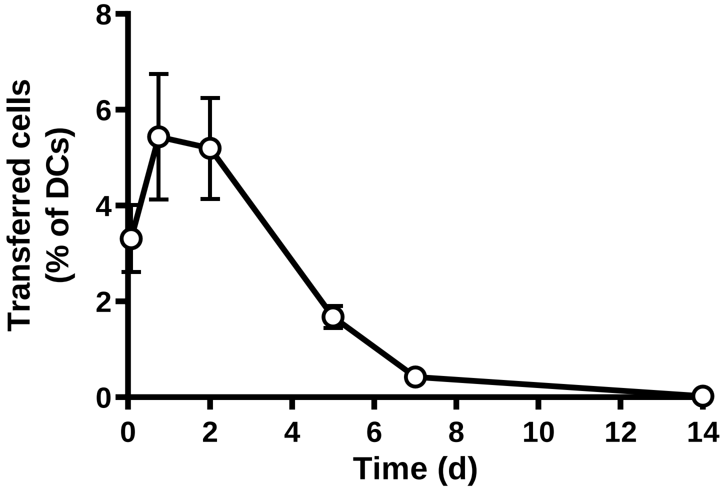

Supplementary Fig. 5 Timeline of the percentage of adoptively transferred DCs in the thymus.

Homing of 1×10^7 adoptively transferred, splenic, in vivo Flt3L expanded DCs from WT mice into CD45 congenic recipient mice was assessed by flow cytometry. The percentage of homed DCs among all DCs in the thymus was evaluated at the indicated time points post transfer. Bars and error bars represent mean ± SEM. n = 5 animals (5 days and 14 days) and n = 10 animals (2hrs, 18 hrs, 2 days and 7 days)

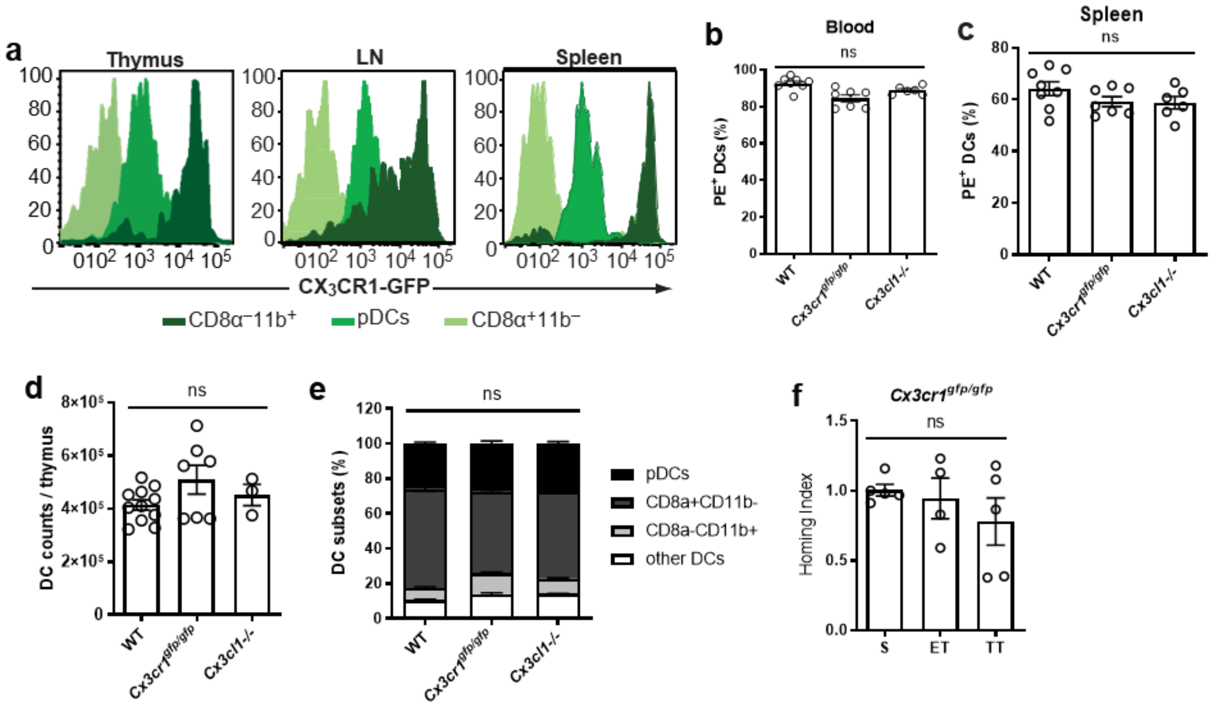

Supplementary Fig. 6 Characterization of *Cx3cr1*$^{gfp/+}$ and *Cx3cl1*$^{-/-}$ mice.

(a) CX_3CR1 expression on DC subsets was evaluated by flow cytometry using *Cx3cr1*$^{gfp/+}$ reporter mice. The representative histograms show the CX_3CR1-GFP expression in CD8α$^+$CD11b$^-$ (light green), CD8α$^-$CD11b$^+$ dendritic cells (DCs, dark green) and plasmacytoid dendritic cells (pDCs, mid-green) of *Cx3cr1*$^{gfp/+}$ animals in thymus (left panel), lymph node (LN, middle panel) and spleen (right panel). n = 3 experiment with 5 mice each. (b and c) WT (n=15), *Cx3cr1*$^{gfp/gfp}$ (n=7) and *Cx3cl1*$^{-/-}$ (n=3) were injected IV with anti-CD11c-PE, sacrificed 2 minutes later and PE+ DCs in blood (b) and spleen (c) were analyzed by flow cytometry (one-way ANOVA), n.s.: not significant. (d) Absolute cell numbers of whole thymi of sex and age matched WT, *Cx3cr1*$^{gfp/gfp}$ and *Cx3cl1*$^{-/-}$ mice. n = 3 independent experiments with 3 mice (one-way ANOVA), n.s.: not significant. (e) Subset composition of thymic DCs in sex and age matched WT, *Cx3cr1*$^{gfp/gfp}$ and Cx3cl1$^{-/-}$ mice. n = 3 independent experiments with 3 mice (two-way ANOVA), n.s.: not significant. (f) Recruitment of circulating WT and *Cx3cr1*$^{gfp/gfp}$ DCs to spleen (S), TT and ET was assessed by competitive homing assays. A 1:1 mixture of differentially labeled splenic, in vivo Flt3L expanded DCs from WT and *Cx3cr1*$^{gfp/gfp}$ donors was injected IV into mice that were previously transplanted with a fetal thymus. The homing index was determined after 18h by FACS analysis of the indicated recipient tissue. Results were pooled from 2 independent experiments, n = 5 mice/group (one-way ANOVA). Bars and error bars represent mean ± SEM. n.s.: not significant.

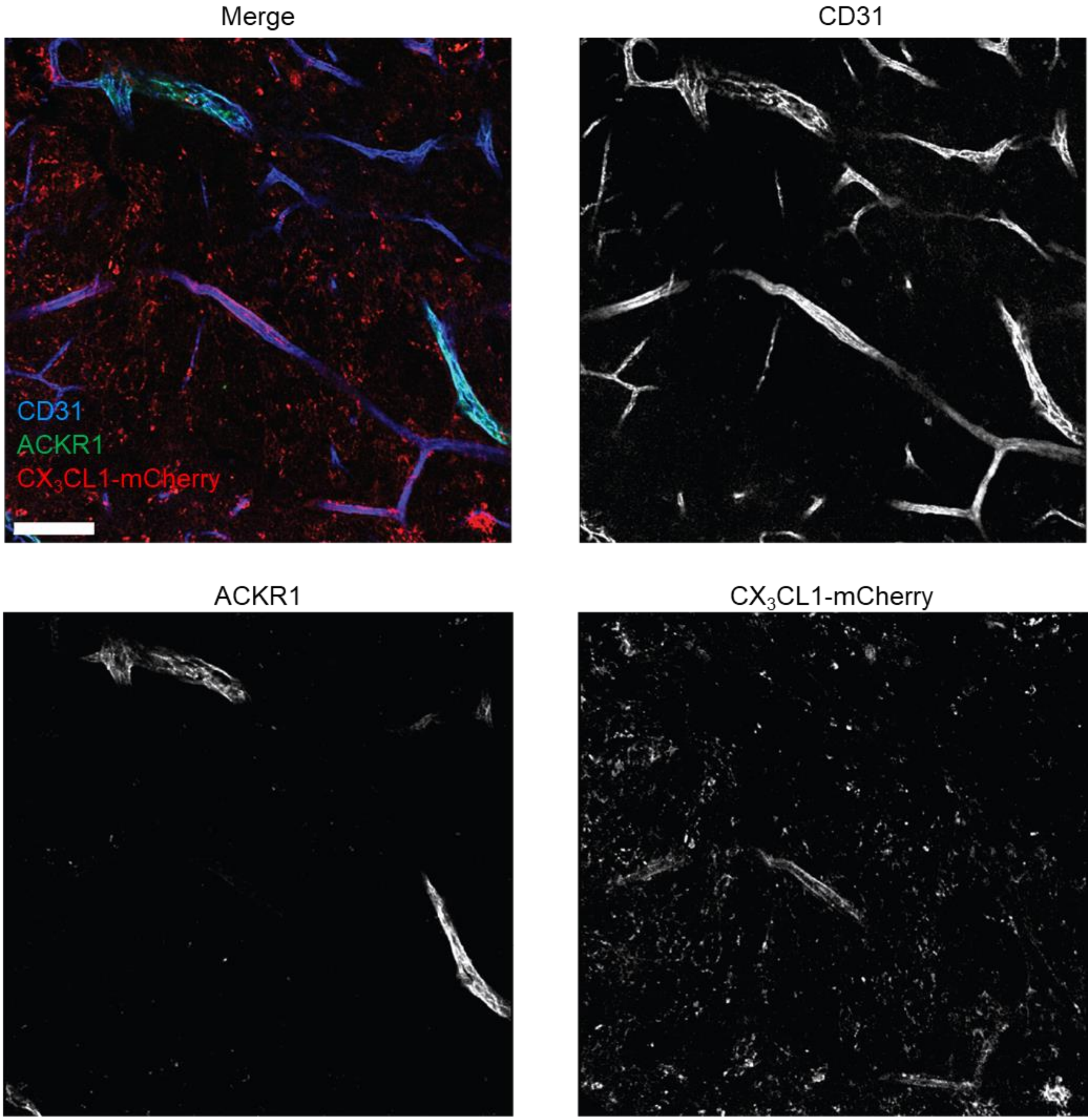

Supplementary Fig. 7 Thymic expression pattern of CX_3CL1.
Representative confocal micrograph of three ETs from CX_3CL1-mCherry reporter mice shows the expression of CD31 (white), ACKR1 (green) and CX_3CL1 (red). The scale bar indicates 100 µm.

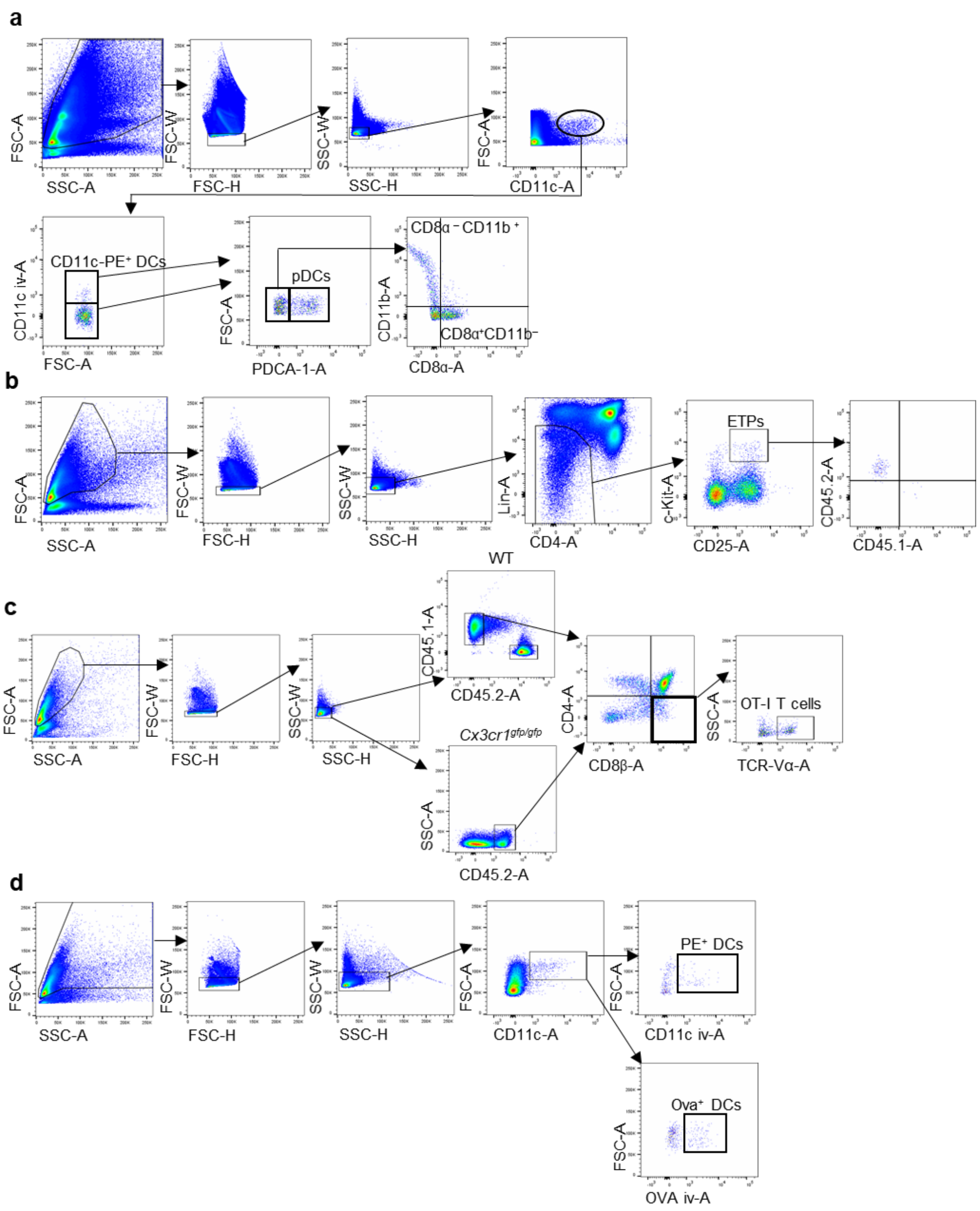

Supplementary Figure 8. Gating strategies used for phenotypic analysis

(a) pDCs were identified as single cells that express CD11c and PDCA-1. CD8α$^-$CD11b$^+$ and CD8α$^+$CD11b$^-$ DCs were gated from the PDCA-1 negative population. (b) ETPs in parabiotic mice were gated as single cells that are Lin$^-$ (Gr-1, CD11b, B220, CD3, NK1.1, Terr119) CD4$^-$ and express c-Kit and CD25. The percent of endogenous vs partner derived ETPs was calculated based on the CD45.1 and CD45.2 expression. (c) OT-I thymocytes in in vivo deletion experiments were detected by gating on single cells that are CD45.1$^+$CD45.2$^-$ that express TCR Vα for CX$_3$CR1 sufficient animals or CD45.2$^+$ that express TCR Vα for *Cx3cr1$^{gfp/gfp}$* animals. (d) BLT mice were either injected with CD11c-PE, isotype-PE or Ova-AF488, respectively. Human dendritic cells were analyzed by gating on CD11c$^+$ single cells and then evaluation of positivity of foe PE or AF488, respectively.

Antibody	Clone	Vendor	Catalogue number	dilution factor	Format / conjugate
Anti- human CD11c	3.9	Biolegend	301622	100	Alexa Fluor® 647
Anti- human CD11c	Bu15	Biolegend	337218	100	APC/Cyanine7
Anti- human CD11c	3.9	Biolegend	301606	200	PE
Anti- human CD31	WM59	Biolegend	303110	100	Alexa Fluor® 488
Anti- human CD31	WM59	Biolegend	303112	100	Alexa Fluor® 647
Anti- mouse B220	RA3-6B2	BioLegend	103226	400	Alexa Fluor® 647
Anti- mouse B220	RA3-6B2	BioLegend	103224	400	APC/Cyanine7
Anti- mouse B220	RA3-6B2	BioLegend	103206	400	FITC
Anti- mouse B220	RA3-6B2	BioLegend	103208	400	PE
Anti- mouse B220	RA3-6B2	BioLegend	103222	400	PE/Cyanine7
Anti- mouse B220	RA3-6B2	BioLegend	103236	400	PerCP/Cyanine5.5
Anti- mouse CD11c	N418	BioLegend	117313	100	Alexa Fluor® 488
Anti- mouse CD11c	N418	BioLegend	117312	400	Alexa Fluor® 647
Anti- mouse CD11c	HL3	BD Biosciences	553801	400	FITC
Anti- mouse CD11c	HL3	BD Biosciences	557401	400	PE
Anti- mouse CD11c	N418	BioLegend	117308	400	PE
Anti- mouse CD11c	N418	BioLegend	117318	400	PE/Cyanine7
Anti- mouse CD11c	HL3	BD Biosciences	561022	400	PE/Cyanine7
Anti- mouse CD24	M1/69	BD Biosciences	553260	200	Biotin
Anti- mouse CD24	30-F1	BioLegend	138504	400	PE
Anti- mouse CD25	PC61	BioLegend	102008	400	PE
Anti- mouse CD26	H194-112	BioLegend	137806	200	FITC
Anti- mouse CD3	17A2	BioLegend	100206	400	PE
Anti- mouse CD31	390	BioLegend	102414	100	Alexa Fluor® 488
Anti- mouse CD31	390	BioLegend	102416	100	Alexa Fluor® 647
Anti- mouse CD4	GK1.5	BioLegend	100412	400	APC
Anti- mouse CD4	GK1.5	BioLegend	100422	400	PE/Cyanine7
Anti- mouse CD4	RM4-5	BioLegend	100540	400	PerCP/Cyanine5.5
Anti- mouse CD45	30-F11	BioLegend	103106	400	PE
Anti- mouse CD45	30-F11	BioLegend	103114	400	PE/Cyanine7
Anti- mouse CD45.1	A20	BioLegend	110708	400	PE
Anti- mouse CD45.1	A20	BioLegend	110730	400	PE/Cyanine7
Anti- mouse CD45.2	104	BioLegend	109824	400	APC/Cyanine7
Anti- mouse CD45.2	104	eBioscience	47-0454	400	APC-eFluor 780
Anti- mouse CD45.2	104	BioLegend	109828	400	PerCP/Cyanine5.5
Anti- mouse CD62L	MEL-14	BioLegend	104421	400	Alexa Fluor® 647
Anti- mouse CD69	MEL-14	BioLegend	104412	400	APC
Anti- mouse CD8α	53-6.7	BioLegend	100714	400	APC/Cyanine7
Anti- mouse CD8α	53-6.7	BioLegend	100706	400	FITC
Anti- mouse CD8α	53-6.7	BioLegend	100734	400	PerCP/Cyanine5.5

Anti- mouse CD8β	YTS156.7.7	BioLegend	126612	400	Alexa Fluor® 647
Anti- mouse c-Kit	2B8	eBioscience	47-1171-82	200	APC-eFluor 780
Anti- mouse c-Kit	2B8	eBioscience	12-1171-82	200	FITC
Anti- mouse EpCAM	G8.8	BioLegend	118212	100	Alexa Fluor® 647
Anti- mouse Flt3	A2F10	BioLegend	135308	200	Biotin
Anti- mouse Gr-1	RB6-8C5	BioLegend	108407	400	PE
Anti- mouse Ly51	6C3/BP-1	BioLegend	108308	200	PE
Anti- mouse MHCII	M5/114.15.2	BD Biosciences	107628	200	APC/Cyanine7
Anti- mouse NK1.1	PK136	BioLegend	108708	400	PE
Anti- mouse PDCA-1	927	BioLegend	127014	400	Alexa Fluor® 647
Anti- mouse PDCA-1	129C1	BioLegend	127106	400	Alexa Fluor® 647
Anti- mouse PDCA-1	927	BioLegend	127006	400	Biotin
Anti- mouse PDCA-1	927	BioLegend	127010	400	PE
Anti- mouse PDCA-1	927	eBioscience	46-3172-82	400	PerCP/Cyanine5.5
Anti- mouse TCR Vα2	B20.1	BioLegend	127812	400	Alexa Fluor® 647
Anti- mouse TCR Vα2	B20.1	BioLegend	127808	400	PE
Anti- mouse TCR Vα2	B20.1	BioLegend	127814	400	PerCP/Cyanine5.5
Anti- mouse Ter119	TER-119	BD Biosciences	553673	400	PE
Anti- mouse VCAM-1	MK2.7	BioXcell	BE0027	100	
Anti- mouse αL integrin	M17/4	BioXcell	BE0006	100	
Anti- mouse/human CD11b	M1/70	BioLegend	101217	400	Alexa Fluor® 488
Anti- mouse/human CD11b	M1/70	BioLegend	101218	400	Alexa Fluor® 647
Anti- mouse/human CD11b	M1/70	BioLegend	101216	400	PE/Cyanine7
Anti- mouse/human CD11b	M1/70	BioLegend	101228	400	PerCP/Cyanine5.5
Anti- mouse/human α4 integrin	PS/2	BioXcell	BE0071	100	
hamster IgG and purified neutralizing mAbs to mouse P-selectin	RB40.34	BD Biosciences	553742	100	
InVivoMAb anti-mouse CD16/CD32	2.4G2	BioXcell	BE0307	100	
Streptavidin		BioLegend	405204	400	PE
Streptavidin		BioLegend	405214	400	PerCP/Cyanine5.5

Supplementary table 1. List of antibodies

Commercially available unconjugated and fluorochrome-conjugated antibodies are listed, providing information on the antibody specificity, clone, vendor, catalogue number, used dilution factor and the format / conjugate for each antibody.
