# Supplementary material for: Specialized transendothelial dendritic cells mediate thymic T-cell selection against blood-borne macromolecules
Source: Nat Commun. 2021 Oct 28;12:6230. doi: 10.1038/s41467-021-26446-x (PMC8553756; doi:10.1038/s41467-021-26446-x)
Supplement: Supplementary file 3 — Reporting Summary [file 41467_2021_26446_MOESM3_ESM.pdf]

Corresponding author(s): Ulrich H. von Andrian

Last updated by author(s): 2021/09/02

Reporting Summary

Nature Research wishes to improve the reproducibility of the work that we publish. This form provides structure for consistency and transparency in reporting. For further information on Nature Research policies, see our Editorial Policies and the Editorial Policy Checklist.

Statistics

For all statistical analyses, confirm that the following items are present in the figure legend, table legend, main text, or Methods section.

n/a	Confirmed	
☐	☒	The exact sample size (*n*) for each experimental group/condition, given as a discrete number and unit of measurement
☐	☒	A statement on whether measurements were taken from distinct samples or whether the same sample was measured repeatedly
☐	☒	The statistical test(s) used AND whether they are one- or two-sided<br>*Only common tests should be described solely by name; describe more complex techniques in the Methods section.*
☐	☒	A description of all covariates tested
☐	☒	A description of any assumptions or corrections, such as tests of normality and adjustment for multiple comparisons
☐	☒	A full description of the statistical parameters including central tendency (e.g. means) or other basic estimates (e.g. regression coefficient) AND variation (e.g. standard deviation) or associated estimates of uncertainty (e.g. confidence intervals)
☐	☒	For null hypothesis testing, the test statistic (e.g. *F*, *t*, *r*) with confidence intervals, effect sizes, degrees of freedom and *P* value noted<br>*Give P values as exact values whenever suitable.*
☒	☐	For Bayesian analysis, information on the choice of priors and Markov chain Monte Carlo settings
☐	☒	For hierarchical and complex designs, identification of the appropriate level for tests and full reporting of outcomes
☒	☐	Estimates of effect sizes (e.g. Cohen's *d*, Pearson's *r*), indicating how they were calculated

Our web collection on statistics for biologists contains articles on many of the points above.

Software and code

Policy information about availability of computer code

Data collection	No commercial, open source or custom code was used to collect the data in this study.
Data analysis	For data analysis Flowjo version 10.6.1 and newer, Imaris version 9.5.0 were used, MatLab, GraphPad Prism version 8.1.1 and Microsoft Excel (Office 364) were used.

For manuscripts utilizing custom algorithms or software that are central to the research but not yet described in published literature, software must be made available to editors and reviewers. We strongly encourage code deposition in a community repository (e.g. GitHub). See the Nature Research guidelines for submitting code & software for further information.

Data

Policy information about availability of data

All manuscripts must include a data availability statement. This statement should provide the following information, where applicable:

- Accession codes, unique identifiers, or web links for publicly available datasets
- A list of figures that have associated raw data
- A description of any restrictions on data availability

The data that support the findings of this study are available from the corresponding author upon reasonable request.

In our study we do not generate large scale omics datasets. In our analysis we did not use data that have been obtained from publicly available sources.
The prism files used for data analysis and display can be made available upon request and the corresponding data are provided to the editor in the source data file.

Field-specific reporting

Please select the one below that is the best fit for your research. If you are not sure, read the appropriate sections before making your selection.

[x] Life sciences   [ ] Behavioural & social sciences   [ ] Ecological, evolutionary & environmental sciences

For a reference copy of the document with all sections, see nature.com/documents/nr-reporting-summary-flat.pdf

Life sciences study design

All studies must disclose on these points even when the disclosure is negative.

Sample size	No sample-size calculation was performed. The sample sizes were chosen based on experience in animal experiments and data variability. For in vitro experiments, technical and biological replicates were performed. In animal experiments, each data point indicates an individual animal (biological replicate). Each in vivo experiment has been repeated at least once as an independent experiments on different days using multiple animals (biological replicates) per group. Details on the numbers of replicates and mice used are listed in each Figure legend.
Data exclusions	No data was excluded from the analysis.
Replication	All experiments were performed in independent replicates and could be replicated. Details on the numbers of replicates and mice used are listed in each Figure legend.
Randomization	Data and animals were grouped based on cell types, genotypes or treatment condidtions. No random allocation was used in the study.
Blinding	Labeling of the tubes during sample processing was done using numbering systems. No group, cell type, genotype or treatment condition based labeling was used during the experiment.

Reporting for specific materials, systems and methods

We require information from authors about some types of materials, experimental systems and methods used in many studies. Here, indicate whether each material, system or method listed is relevant to your study. If you are not sure if a list item applies to your research, read the appropriate section before selecting a response.

Materials & experimental systems

n/a	Involved in the study	
[ ]	[x]	Antibodies
[x]	[ ]	Eukaryotic cell lines
[x]	[ ]	Palaeontology and archaeology
[ ]	[x]	Animals and other organisms
[ ]	[x]	Human research participants
[x]	[ ]	Clinical data
[x]	[ ]	Dual use research of concern

Methods

n/a	Involved in the study	
[x]	[ ]	ChIP-seq
[ ]	[x]	Flow cytometry
[x]	[ ]	MRI-based neuroimaging

Antibodies

Antibodies used	Please see supplemental table for full list of antibodies used.<br>Unconjugated and fluorochrome-conjugated Abs to mouse B220 (RA3-6B2), CD4 (GK1.5), CD8α (53-6.7), CD8β (53-5.8), CD11b (M1/70), CD11c (HL3 and N418), CD31 (390 an MEC13.3), CD45.1 (A20), CD45.2 (104), CD69 (H1.2F3), PDCA-1 (927 and 129C1), TCR Vα2 (B20.1), Fc-receptors (2.4G2), hamster IgG and purified neutralizing mAbs to mouse P-selectin (RB40.34), αL integrin (M17/4), α4 integrin (PS2/) and VCAM-1 (MK2.7) were purchased from BD Biosciences, BioLegend, eBioscience and Bio X cell. Chimeric, non-depleting, anti-α4 integrin mAb (CRL19.11) was provided by R. Palframan. Anti-human CD11c (3.9, Bu15, B-ly6 and S-HCL-3) and CD31 (WM59) mAbs were purchased from BioLegend and BD Biosciences. Anti-cytokeratin 8 mAb secreting hybridoma (TROMA-1) was obtained from the Developmental Studies Hybridoma Bank at the University of Iowa and PTX was from Calbiochem. Alexa Fluor® 488, Alexa Fluor® 647 labeled ovalbumin was purchased from Invitrogen and unlabeled EndoGrade ovalbumin was from Profos. High molecular weight FITC and TRITC dextran (MW = 2×106 Da) were from Invitrogen. Fluorescently labelled Ulex Europaeus Agglutinin I (UEA I) was purchased from Vector Laboratories.
Validation	All antibodies were validated for their antigen specificity on relevant species and used according to the manufacturer's instructions. Antibodies were titrated prior to use for optimal signal to noise ratio. All antibodies have been validated and reported in prior studies.

Animals and other organisms

Policy information about studies involving animals; ARRIVE guidelines recommended for reporting animal research

Laboratory animals	Unless otherwise stated, all animals used in this study were female, 4-12 weeks of age.<br>C57BL/6 mice were from Charles River Laboratories and were used at 4–12 weeks of age. OT-I mice, which carry a transgenic TCR specific for ovalbumin amino acids 257–264 (SIINFEKL) in H-2Kb, were from Taconic Farms. Cx3cr1gfp/gfp x CD45.1, Cx3cr1gfp/gfp x CD45.2 and CD11c-YFP mice were purchased from the Jackson Laboratory are described elsewhere. Cx3cr1gfp/gfp x OT-I x CD45.2 mice were generated in the lab. Cx3cl1–/–and Cx3cl1-mCherry mice were a gift from A Lira and S Jung, respectively. NOD/SCID/IL2Rγ –/– (NODSCIDGAMMA - NSG) mice (The Jackson Laboratory) were used for BLT mice generation. 6- to 8-week old NSG mice were used.<br>Mice were housed in 12/12 hrs light/dark cycles (6 am to 6pm light) at a 70~72 F degree temperature range, and relative humidity within 40~50% .
Wild animals	The study did not involve wild animals.
Field-collected samples	The study did not involve samples collected from the field.
Ethics oversight	Anonymized human fetal tissues were acquired through ABR (Alameda, CA) and utilized under a Massachusetts General Hospital Institutional Review Board-approved protocol. All humanized mouse experiments were approved by the Massachusetts General Hospital Institutional Animal Care and Use Committee (IACUC) under the approved protocol 2009N000136. Experiments were carried out in accordance with the recommendations in the Guide for the Care and Use of Laboratory Animals of the National Institutes of Health.

Note that full information on the approval of the study protocol must also be provided in the manuscript.

Human research participants

Policy information about studies involving human research participants

Population characteristics	Anonymized human fetal tissues were acquired through ABR (Alameda, CA) and utilized under a Massachusetts General Hospital Institutional Review Board-approved protocol.
Recruitment	Anonymized human fetal tissues were acquired through ABR (Alameda, CA) and utilized under a Massachusetts General Hospital Institutional Review Board-approved protocol. All the human tissue used in the experiment for the implantation in the BLT mice was obtained from consenting donors. Consent forms are obtained by the clinics performing the procedure. MGH IRB Committee approved the use of human tissue. The IRB approval form is submitted with our mansucript.
Ethics oversight	All humanized mouse experiments were approved by the Massachusetts General Hospital Institutional Animal Care and Use Committee (IACUC) under the approved protocol 2009N000136.

Note that full information on the approval of the study protocol must also be provided in the manuscript.

Flow Cytometry

Plots

Confirm that:

- [x] The axis labels state the marker and fluorochrome used (e.g. CD4-FITC).
- [x] The axis scales are clearly visible. Include numbers along axes only for bottom left plot of group (a 'group' is an analysis of identical markers).
- [x] All plots are contour plots with outliers or pseudocolor plots.
- [x] A numerical value for number of cells or percentage (with statistics) is provided.

Methodology

Sample preparation	Cell preparation. To analyze DCs in thymi, LNs and spleens, the organs were harvested and digested in 50 µg/ml (for thymi and LNs) or 100 µg/ml Liberase TM (for spleens; Roche) for 20 min at 37ºC. Single cell suspensions for analysis of lymphocytes in thymi, LNs and spleens were generated by mechanical organ dissociation. Blood samples were obtained by retroobital bleeding followed by red blood cell lysis in ACK buffer for 60 seconds. Cells were stained for surface markers and analyzed on a FACS Canto (BD Biosciences). For cell sorting, thymi were digested in 50 µg/ml Liberase TM (Roche) for 20 min at 37ºC followed by density gradient centrifugation. Thymic stromal cells were prepared by centrifugation over Percoll (GE Healthcare) as described previously REF13 and DCs by centrifugation over NycoPrep (Axis-Shield) according to the vendor’s manual.<br>Homing assays. Donor DCs were isolated from C57BL/6 mice that had been injected subcutaneously with 2 × 106 to 5 × 106 B16 melanoma cells secreting Flt3 ligand as described 85. After 10–14 d, mice were killed and splenic DCs were purified by digestion in 100 µg/ml Liberase TM (Roche) for 20 min at 37ºC followed by density-gradient centrifugation over NycoPrep (Axis-Shield) according to the vendor’s manual. These preparations routinely contained 75–85% CD11c+ DCs. When indicated, DC maturation was induced by culture for 24 h in complete medium in the presence of 1 µg/ml of lipopolysaccharide (E. coli 0.26:B6; Sigma-Aldrich). Mature DC cultures typically resulted in enrichment in CD11c+ cells (90–95%) and in the up-regulation of classical maturation markers (CD86 and MHC class II) for all CD11c+ cells. Immature or

	mature DCs were labeled for 15 min at 37ºC with 30 µM CFSE (carboxyfluorescein succinimidyl ester) or 3 µM DDAO-SE (7-hydroxy-9H-(1,3-dichloro-9,9-dimethylacridin-2-one) succinimidyl ester (all from Molecular Probes) at a cell concentration of 107 cells/ml. Dead cells and excess label were removed by centrifugation, and 1×107 labeled DCs were then injected in the tail veins of recipient mice. In some experiments, DCs from Cx3cr1gfp/gfp mice were isolated or WT DCs were pretreated with 50 µg/ml of blocking mAb or PTX (Calbiochem) for one hour at 37ºC and washed before simultaneous injected with control populations; for inhibition of endothelial adhesion molecules, 100 µg mAb was injected along with the labeled DCs. Mice were killed at indicated time points and single-cell suspensions were generated from spleens and thymi and cell samples were analyzed by flow cytometry. The total number of homed DCs was calculated by multiplication of the fraction of CFSE+ (or DDAO-SE+) CD11c+ events by the total cellularity of the target organ.
Instrument	Homing of DC subsets and stability of DC subsets was addressed by transfer of DC subsets that had been sorted on a FACS Aria II (BD Bioscience) followed by fluorescent labeling with CFSE, DDAO-SE and CMTMR, respectively (all from Molecular Probes) and intravenous injection into recipient mice. Mice were sacrificed and homed DCs were analyzed for their respective subset 18 h after transfer on an LSR II (BD Biosciences).
Software	For data analysis Flowjo version 10.6.1 was used.
Cell population abundance	Cell population abundance is shown and quantified in the figures.
Gating strategy	All experiments were gated on FSC-A/SSC-A, doublet discrimination using SSC-H/SSC-W and FSC-H/FSC-W. CD11c+ were identified via CD11c+/SSC-A, DC subsets were gated on CD11b+ vs CD8a for CD8a+CD11b- and CD8a-CD11b+ DC subsets. pDCs were identified as CD11c+ PDCA-21+. OT-I T cells were gated as FSC-A/SSC-A, doublet discrimination using SSC-H/SSC-W and FSC-H/FSC-W, CD45.1/CD45.2, CD4/CD8a, CD8+ TCRVa2+.

☒ Tick this box to confirm that a figure exemplifying the gating strategy is provided in the Supplementary Information.
