# Supplementary material for: Specialized transendothelial dendritic cells mediate thymic T-cell selection against blood-borne macromolecules
Source: Nat Commun. 2021 Oct 28;12:6230. doi: 10.1038/s41467-021-26446-x (PMC8553756; doi:10.1038/s41467-021-26446-x)
Supplement: Supplementary file 4 — Description of Additional Supplementary Files [file 41467_2021_26446_MOESM4_ESM.pdf]

Description of Additional Supplementary Files

Supplementary Movie 1. Motility of endogenous DCs and homed DCs 2 h post transfer.

Transplanted, BM chimeric animal that has received mixed BM from CD11c-YFP and WT donors at a ratio of 1:1 was subjected to MP IVM 2 h after transfer of CMAC labeled DCs. The lumen of the vasculature was visualized by IV injection of TRITC-dextran (red). Endogenous DCs are depicted in green and homed DCs in blue. Images were acquired every 20 s in the z dimension over a distance of 42 μm. Scale bar is 50 μm. Video playback is at 15 frames per second (time-lapse 300×).

Supplementary Movie 2. Motility of endogenous DCs and homed DCs 18 h post transfer.

MP IVM 18 h after transfer of CM-TMR labeled DCs. The lumen of the vasculature was visualized by IV injection of Qtracker® 655 vascular label (magenta). Endogenous DCs are depicted in green and homed DCs in red. Images were acquired every 18 s in the z dimension over a distance of 30 μm. Scale bar is 50 μm. Video playback is at 15 frames per second (time-lapse 270×).

Supplementary Movie 3. DCs in close contact with the thymic vasculature.

Transplanted, BM chimeric animal that had received mixed BM from CD11c-YFP and WT donors was subjected to MP IVM. The lumen of the vasculature was visualized by IV injection of Qtracker® 655 vascular label (magenta). DCs are depicted in green. The corrected image acquisition rate was 21.3 s in the z dimension over a distance of 32 μm. Scale bar is 20 μm. The movie is a compilation of a standard video playback, followed by a surface-rendered rotation of the same video, followed by a 3D reconstruction that was cropped in the middle to visualize intravascular parts of the depicted DC.

Supplementary Movie 4. 3D rendering of TE-DC protrusions.

A fixed tissue section obtained from the transplanted BM chimeric animal described in Suppl. Movie 3 was analyzed by confocal single-photon excitation microscopy. The confocal z-stack obtained was processed post-acquisition to obtain a 3D surface rendering animation. In the video, the vasculature corresponds to CD31 staining (blue), perivascular $CD11c^+$ DCs are shown in green (GFP signal) and their intravascular protrusions have been pseudocolored in red.

Supplementary Movie 5. Analysis of an intravascular protrusion at high magnification. Zoomed-in detail of one of the protrusions observed in the 3D surface rendering shown in Movie 4, using same colors for the different cells and anatomical structures depicted. The video highlights the cell body constriction experienced by a perivascular DC at the site of insertion into and across the endothelial layer to successfully protrude towards the vascular lumen.
